# Anti-proliferative activity of *Artemisia marschalliana* on cancerous cell lines

**DOI:** 10.1186/s12906-023-03887-z

**Published:** 2023-04-14

**Authors:** Haleh Forouhandeh, Vahideh Tarhriz, Masoumeh Zadehkamand, Parina Asgharian

**Affiliations:** 1 Molecular Medicine Research Center, Biomedicine Institute, Tabriz University of Medical Sciences, Tabriz, Iran; 2 Student Research Committee, Faculty of Pharmacy, Tabriz University of Medical Sciences, Tabriz, Iran; 3 Immunology Research Center, Tabriz University of Medical Sciences, Tabriz, Iran; 4 Drug applied research center,, Tabriz University of Medical Sciences, Tabriz, Iran

**Keywords:** *Artemisia marschalliana*, Cytotoxic activity, Apoptosis, Cancerous cells

## Abstract

**Background:**

The genus *Artemisia* of the *Asteraceae* family has different species that are used in the treatment of a wide range of diseases, including cancers due to the presence of valuable compounds and important medicinal properties. Various studies on the anti-tumor effect of different species of *Artemisia* have proven the cytotoxic properties of these plants in cancer treatment, and several anti-cancer compounds of this genus have been purified.

**Objective:**

The objective of this study was to investigate the cytotoxicity and related mortality mechanisms of *Artemisia marschalliana* essential oil and extracts.

**Methods:**

The essential oil and various extracts of *Artemisia marschalliana* were elicited using a Soxhlet extractor. Anti-cancer to anti-proliferative activity as MTT assay is measuring cancerous and non-cancerous cell viability. In the next step, the strongest extract fractions were obtained by using the vacuum liquid chromatography method. Flow cytometry was applied to identify the mechanism of cell death, and a Real-time polymerase chain reaction test of apoptosis genes, which encode apoptosis-regulating proteins, was measured to confirm the flow cytometry results.

**Results:**

The strongest extract belonged to dichloromethane extract 60% fraction of the extract on breast cancer cells and 80% fraction on liposarcoma cancer cells showed the most cytotoxicity within 48 h, while, the fractions did not notable cytotoxicity of non-cancerous cells cell. Flow cytometry analysis illustrated the mentioned extract and its fractions kill cancer cell lines through the apoptosis mechanism. Our findings confirmed the flow cytometry results. In addition, the essential oil of *Artemisia marschalliana* showed a considerable cytotoxic property.

**Conclusion:**

Dichloromethane extract of *Artemisia marschalliana* shoot and its 60 and 80% fraction selectively inhibited the growth of cancer cells by inducing the apoptosis mechanism. Regarding obtained results, 60 and 80% fractions of dichloromethane extract can be a good candidate for future studies in the field of identification and separation of pure cytotoxic compounds.

## Introduction

Cancer is the abnormal growth of cells that can invade and metastasize to other parts of the body [1, 2]. The disease is still one of the leading causes of death worldwide [3]. In 2022, 1,918,030 new cases of cancer and 609,360 cancer deaths are projected in the United States [4]. It is estimated that one-third of cancer death is due to five important risk factors including high BMI (Body mass index), not consuming fruits and vegetables, low physical activity, tobacco, and alcohol consumption [5]. Liposarcoma (LS) is a common type of soft tissue sarcoma [6, 7]. In addition, breast cancer is one of the most important and common malignancies in Iranian women [8]. The type of cancer and the rate of its progression will determine the treatment methods. Surgery, chemotherapy, radiotherapy, immunotherapy, gene therapy, cell therapy, and hormone therapy are common methods to treat cancer. However, these treatments face limitations [9]. Medicinal plants have long been the most important source of treating various diseases. Today, cytotoxic secondary metabolites and their semi-synthetic derivatives have a special place in cancer therapies, therefore, the study of medicinal plants has been considered by researchers around the world [10, 11]. Among the known medicinal plants, the genus *Artemisia* of the family *Asteraceae* has noteworthy compounds for their following effects: antioxidant activity [12], anti-inflammatory properties, antimalarial, antiviral, antibacterial, antifungal, and antitumor effects [13]. It should be noted that the anti-cancer effect of artemisinin and its derivatives has been proven in various studies [14, 15]. Significant antitumor compounds in *Artemisia* include monoterpenes, terpenes, and phenolic compounds [13]. Apoptosis is the predominant effect of extracts or active ingredients of Artemisia species. These species induce apoptosis in different cell lines by activating caspases, depolarizing the mitochondrial membrane potential, and reducing BCL-2 expression [16] or stopping the cell cycle [17, 18]. Hu et al. observed the usual morphological changes of apoptosis, including dense chromatin and volume reduction by exposure to another human hepatoma cell line with components of *Artemisia capillaries* Thunberg [17]. Based on previous reports, *Artemisia* species have various biologically active compounds and different medicinal properties of their unique compounds in this genus. To achieve this goal and considering the presence of valuable compounds in the genus *Artemisia*, the present study investigated to explore the cytotoxic effects of different extracts of *A. marschalliana* on cancer cells (MCF-7 and SW872) and normal cells (MCF-10A).

## Materials and methods

### Preparation of plant samples

Plant *A. marschalliana* (synonym of *Artemisia campestris subspindora*) was collected in September 2016 from the Kalibar region of East Azerbaijan province, Iran, (38°52′04.5″N 47°02′26.4″E). The plant was identified by Dr. Fatemeh Ebrahimi at the herbarium of Tabriz University of Medical Sciences, Tabriz, Iran (Code number: TBZ-FPH 4037). *A. marschalliana* is freely available and used by local people as a plant in traditional medicine and there is no constraint by the authority to collect the plant. The experimental research and field studies on plants, including the collection of plant material, comply with relevant institutional, national, and international guidelines and legislation, which were approved by the Research Ethical Committee (REC) Numbers: IR.TBZMED.RDC.1396.1187 and IR.TBZMED.RDC.1397.012, Tabriz University of Medical Sciences, Tabriz, Iran.

### Preparation of extracts

The shoots of *A. marschalliana* were washed carefully and dried in the open sterile air condition. The dried plants were powdered using an electric mill for subsequent experiments. After the shoots of *A. marschalliana* were dried in a laboratory under an electric mill, 200 g of shoot powder were extracted by Soxhlet apparatus with solvents of n-Hexane (n-Hex), dichloromethane (DCM), and methanol (MeOH). The obtained extracts were dried to remove solvents completely by using a rotary evaporator at 45 °C [19].

### Preparation of DCM extract fraction by VLC method

We filled the VLC hopper with silica gel using a vacuum pump and after washing with 150 mL of methanol and ethyl acetate solvents, loaded 2 g of the plant sample into it. Then, washed the funnel with 200 mL of a mixture of ethyl acetate and hexane solvents with increasing polarity (10, 20, 40, 60, 80, and 100%), and the output of each wash in a separate container. Finally, we poured and rotated them with an evaporator at 45 °C [19].

### Essential oils

First, we poured 150 g of dried and ground plant powder into the jouette balloon, and then poured up to two-thirds of the balloon volume in the ratio of 1 to 3 glycerin and water on the plant powder. To prevent heat loss, we covered the Clevenger tube with heat insulation. The total time of essential oil collection was 4 h and the maximum amount of essential collected oil was in the first hour [20].

### Cell lines used in experiments

Human breast cancer cell lines with estrogen, progesterone, and glucocorticoid receptors (MCF-7), undifferentiated malignant tumors consistent with human liposarcoma cells (SW872) were used as cancerous cell lines and normal human mammary epithelial cells (MCF-10A) was used as the control cells. The cell lines were purchased from the National Cell Bank, Pasteur Institute of Iran.

### Anti-proliferative activity of extracts by MTT assay

The substance MTT (dimethylthiazole-2 and 5-diphenyltetrazolium bromide) turns into insoluble purple formazan crystals due to the cleavage of the tetrazolium ring by mitochondrial enzymes in living cells. Therefore, to determine extracts toxicity on cell lines, and calculate of IC_50_ value, MTT assay was used [21]. RPMI 1640 culture medium with 10% fetal bovine serum (FBS) (Gibco), and 1% penicillin/streptomycin (Gibco), was used for cell culture experiments. To perform this test, 15,000 cells we spread into each well of a 96-plate with a volume of 200 μL. After 24 hours, the cells adhered to the bottom of the plate. The different concentrations of extract including 10, 20, 50, 100, 150, 200, and 300 μg/mL were treated on the well in three series of repetitions. Taxol was considered a reference drug control and Dimethyl sulfoxide (DMSO) as a positive control. The cells were incubated at 37 °C, 5% CO2, and 95% humidity for 24 and 48 hours, and then the culture medium is drained by a sampler, and 50 μL of MTT solution with a concentration of 2 mg/mL and 100 μL of complete culture medium was added to each well. This should be done in very low light, then we wrap the plate in aluminum foil and put it in the incubator for 4 hours. After 4 hours of incubation, the content of the 96-well plate was emptied, and by adding 200 μl of DMSO, the formazan crystals were dissolved and the purple color created by the plate reader was measured at a wavelength of 570 nm [22].

### Detection by flow cytometry

The number of dead cells treated with different extracts via the apoptosis mechanism was determined by flow cytometric assay kit (BD bioscience, NJ, USA) at 488 (excitation wavelength) and 617 nm (emission wavelength) [23, 24]. To perform a flow cytometry test, we added 2 × 10^5^ cells to each 6-cell well plate. Then, added 150 μL of trypsin and dilute with 50 μL of PBS. One hundred microlitre of the Annexin V binding buffer with deionized water (1: 10) with along 5 μL of Annexin V and 5 μL of Propidium Iodide were added to each microtube except for unstained control. Non-color control was used to remove any background adsorption and color control was used to prevent natural cell apoptosis or necrosis from interfering with the results of the samples [24].

### Quantitative RT-PCR analysis and western blotting tests

Total RNA was extracted using RNX-Plus reagent (Cinnagen, Co. Tehran, Iran) from treated and non-treated cells according to the manufacturer protocol, and single-strand cDNAs were synthesized using a random hexamer and oligo dT primers using SinaClon, Cat. No.: RT5201. RT-PCR was carried out for *P53* gene in the presence of specific primers (forward: 5′-CCCATCCTCACCATCATCACAC-3′ and reverse: 5′-GCACAAACACG CACCTCAAAG-3′) and *Bax* gene (forward: 5′-GATGCGTCCACCAAGAAGCT-3′ and reverse: 5′-CGGCCCCAGTTGAAGTTG-3′) [22, 25] using TaKaRa master mix (Cat, No: RR820L) [26]. GAPDH mRNA level was used to normalize the genes under study in the presence of primers (forward, 5′-ATGACTCTACCCACGGCAAG-3′); (reverse, 5′-CTGGAGATGGTGATGGGTT-3′) and the relative quantity of mRNA samples was detected using the standard ΔΔCt method [26, 27]. To confirm RT-PCR results, BAX and BCL-2 protein levels were evaluated in treated and non-treated cells. For this aim, whole cell contents were isolated using a lysis buffer containing 500 μl of Tris-Hcl: 500 μL, EDTA: 3 mg, NaCl: 80 mg, Deoxycholic acid sodium salt (DOS): 25 mg, SDS: 10 mg, and 10 μL of 1% Triton with protease inhibitory cocktail [28, 29]. The concentration of isolated proteins was determined using the Bradford concentration method. Then 12 μl of protein was separated on 5% SDS-PAGE gel to isolate proteins and replace nitrocellulose membrane (PVDF) (Amersham, Cat. No: 10600023) using the semidry electrophoretic transfer method [28, 29]. Blocking solution was then used to prevent non-specific reaction of the initial antibody. Primary antibody in the blocking solution to prevent non-specific reaction added to PVDF and incubated. Furthermore, the secondary antibody was utilized to detect desired protein band [26, 30]. Chemoluminescence kit (ECL™ Advance Blocking Reagent Cytiva, RPN418) was used to visualize the desired protein band [26, 30]. Anti- BAX (B-9): sc-7480, and anti-BCL-2 (N-19): sc-492 in a 1:500 dilution in PBS, 2.5% Blotto, 0.05% Tween-20; β-Actin (C4): sc-47778 1:10,000 dilution in PBS, 2.5% Blotto, 0.05% Tween-20, mouse-IgGκ BP-HRP: sc-516102 1:10,000 in a dilution in PBS, 2.5% Blotto, 0.05% Tween-20 as secondary antibody, and protein ladder- Super Signal® Molecular Weight (MAN0011723) were utilized.

### GC/MS analysis

DCM extract (1 μL) was injected into the apparatus of Shimadzu GCMS-QP5050A equipped with a DB-1 column (60 m × 0.25 mm; film thickness 0.25 μm). The initial temperature (50 °C, 3 min) was raised to 270 °C at a rate of 4 °C/min. The injection temperature was adjusted to 240 °C [31, 32].

### Statistical analysis of data

The half-maximal inhibitory concentration (IC_50_) value of the samples indicated the concentration of the sample, which inhibits 50% of cell growth. *P*-value by ANOVA and Tukey post hoc test analysis in all tests was considered less than 0.05 significant using GraphPad Prism 8.0.1 software.

## Results

### Amounts of extracts and fractions

The weight of obtained extracts as a percentage was shown in Table 1. In addition, the weight of the strongest extract fractions (DCM extract) was also shown as a percentage in Table 1.

**Table 1:** The weight of n-Hex (n-Hexane), DCM (dichloromethane), and MeOH (methanol) extracts powder and fractions of DCM extract from 200 g of *A. Marshalliana*

Methanol extract		Dichloromethane extract		n-Hexan e extract		Extract
10.03		1.25		2.26		*Weight of plant extract (g/100 g of extract)*
**100%**	**80%**	**60%**	**40%**	**20%**	**10%**	*Fractions*
25	10.35	3.05	2.15	–	–	*Weight of DCM extract fraction (g/100 g of extract)*

### Investigation of cytotoxic effects

#### Investigation of cytotoxic effects of the studied plant by MTT assay

**Fig. 1:**
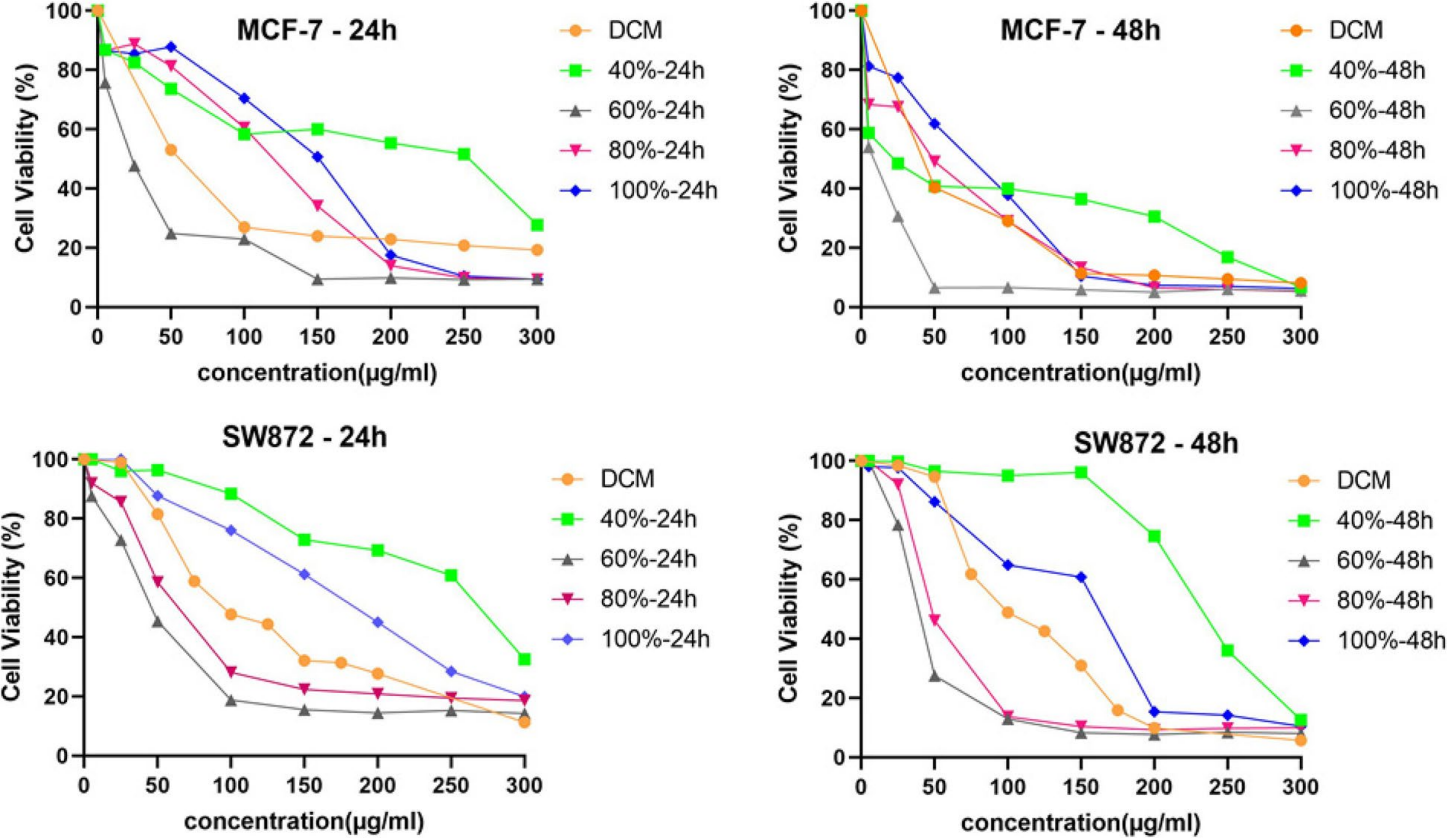
The cell viability of MCF-7 and SW872 cancer cell lines in different concentrations of DCM (dichloromethane) extract and its fractions after **a**) 24 and **b**) 48 hours of incubation

MTT test was performed to determine the anti-proliferative effects of the studied plant. As indicated in Table 2, the low IC_50_ values belonged to DCM extract in both cell lines, which indicated the high anti-proliferative effect of this extract; therefore, DCM extract is fractionated with VLC. Statistical analysis of ANOVA and Tukey post hoc test showed the respective fractions were significantly different from the DMSO control. Data showed that the inhibition of cell growth in both cancer cell lines increases with increasing concentrations of these extracts and fractions, as shown in Fig. 1.

**Table 2:** IC_50_ values of extracts and fractions of the most effective extracts on cancer cells (MCF-7 and SW872) and normal (MCF-10A) after 24 and 48 hours in μg/mL

	MCF-7 (24 h)	MCF-7 (48 h)	SW872 (24 h)	SW872 (48 h)	MCF-10A (24 h)	MCF-10A (48 h)
n-Hexane extract	165.4 ± 8.9	128.1 ± 10.8	132.3 ± 3.6	118.7 ± 8.48	197.7 ± 13.5	86.34 ± 0.4
Dichloromethane extract	41.74 ± 9.2	38.61 ± 0.9	104.4 ± 2.4	102.2 ± 2.1	93.83 ± 0.4	30.7 ± 0.2
Methanol extract	**<** 800	**<** 800	**<** 800	**<** 800	**<** 800	< 800
40% VLC fraction of Dichloromethane extract	199 ± 21.42	39.47 ± 6.08	259.4 ± 48.15	231.3 ± 2.6	142.3 ± 30.11	133.82 ± 12.1
60% VLC fraction of Dichloromethane extract	17.86 ± 2.84	9.42 ± 1.09	43.72 ± 4.4	38.52 ± 4.3	56.67 ± 1.7	63.75 ± 3.7
80% VLC fraction of Dichloromethane extract	136.4 ± 5.3	58.36 ± 3.91	79.94 ± 3.9	69.18 ± 6.7	104.6 ± 6.8	107.2 ± 10.6
100% VLC fraction of Dichloromethane extract	156 ± 0.5	79.39 ± 4.01	179.5 ± 6.2	137.4 ± 6.71	128.9 ± 0.98	134.1 ± 6.29

**Fig. 2:**
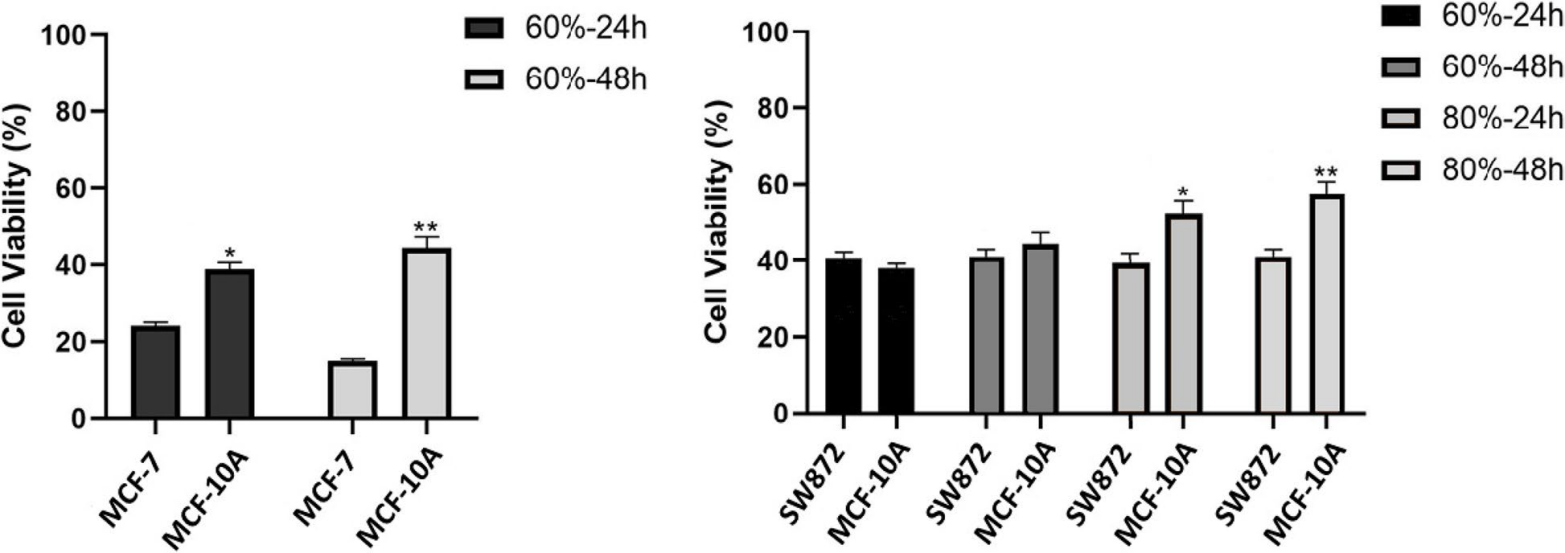
The cell viability of MCF-7 and SW872 cancer cells due to exposure to mentioned fractions of DCM (dichloromethane) extract compared to normal MCF-10A cells

The time-dependent effects of DCM extract and related fractions have been investigated indicated that in the MCF-7 cell line, all plant samples have time-dependent effects, but the effects of cytotoxicity in the SW872 cell line increase significantly only over 40 and 100% fractions over time. The viability of MCF-7 cells in the face of 60% fraction compared to MCF-10A cells is significantly reduced; as a result, it seems that the 60% fraction is the most effective in MCF-7 cell line and had the least adverse effects on normal cells (Fig. 2). 60 and 80% fractions on the SW872 cell line showed the best cytotoxic effect. However, 80% fraction showed a selective cytotoxic effect on the SW872 cell line due to a notable reduction in the viability of SW872 cells compared to MCF-10A cells and was introduced as the best fraction. Furthermore, the essential oil sample of this plant with IC_50_ equivalent to 11.91 ± 2.59 μg/mL inhibited the growth of breast cancer cells. Essential oil yield (w/w_0_) was 74.43%.

#### Apoptosis assay results on MCF-7 and SW872 cells

**Fig. 3:**
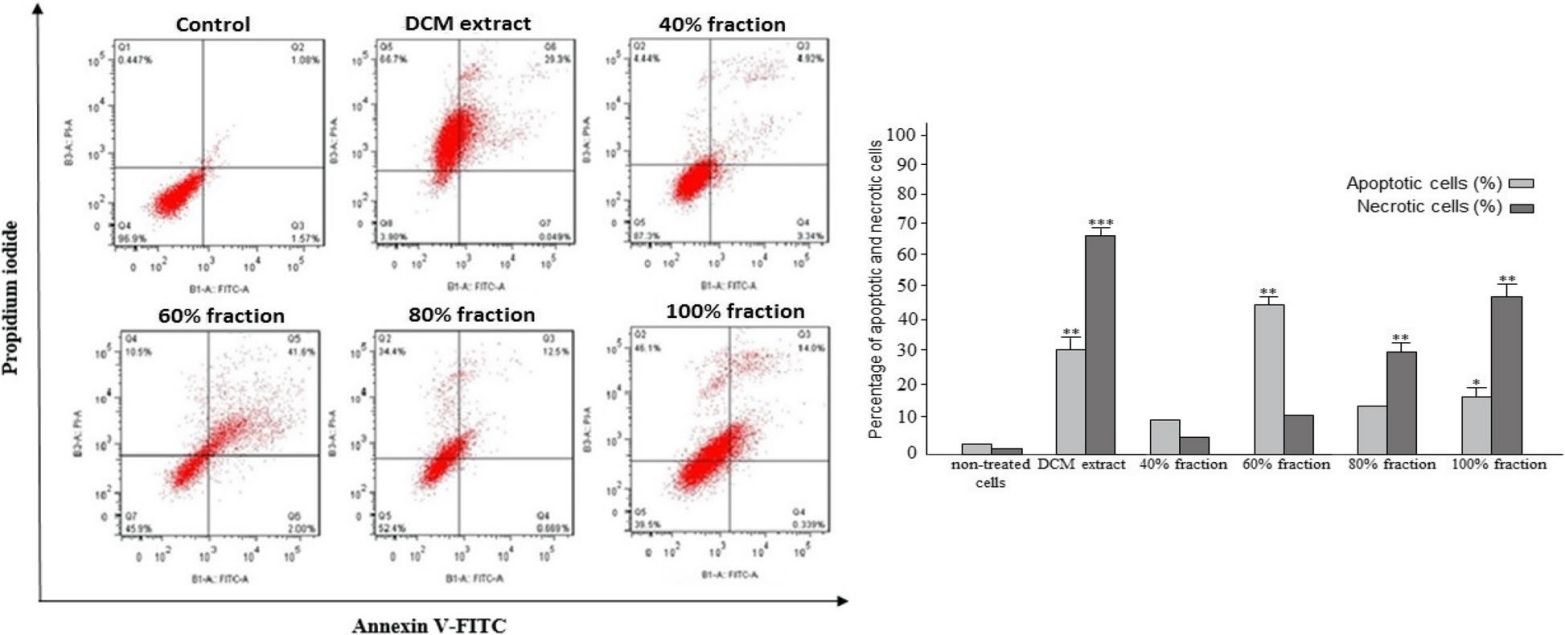
The results of Flow cytometric test against MCF-7 cell line treated with DCM (dichloromethane) extract and its related fraction. The graphs were calculated and drawn using statistical analysis and indicated by an asterisk (*p* ≤ 0.001 ***, *p* ≤ 0.01:**, *p* ≤ 0.05: *)

**Fig. 4:**
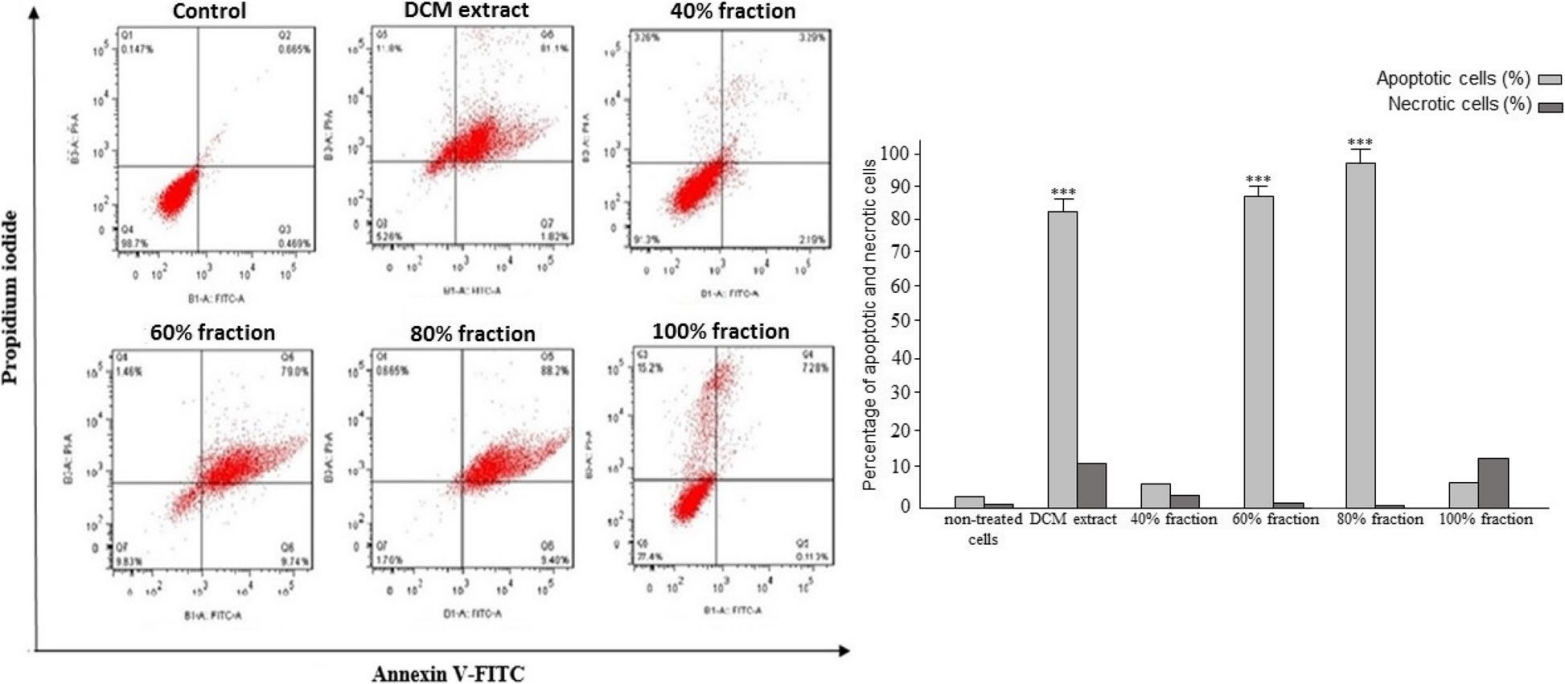
The results of apoptosis assay against SW872 cell line treated with DCM (dichloromethane) extract and its related fractions. The graphs were calculated and drawn using statistical analysis and indicated by an asterisk (p ≤ 0.001 ***, p ≤ 0.01:**, p ≤ 0.05: *)

Flow cytometry tests were utilized to detect the apoptosis rate. The histogram view of apoptosis induction by DCM extract and related fractions in MCF-7 and SW872 cell lines is shown in Figs. 3 and 4. The results revealed that 60% fraction in MCF-7 cell line and DCM extract and 60 and 80% fractions in SW872 cell line had the highest rate apoptosis induction. Moreover, we did not any notable apoptosis or necrosis rate in MCF-10A normal cell line. This valuable finding showed that the effective compounds with anti-cancer features probably belong to the DCM extract and its related fractions.

#### RT-PCR analysis and western blotting results

**Fig. 5:**
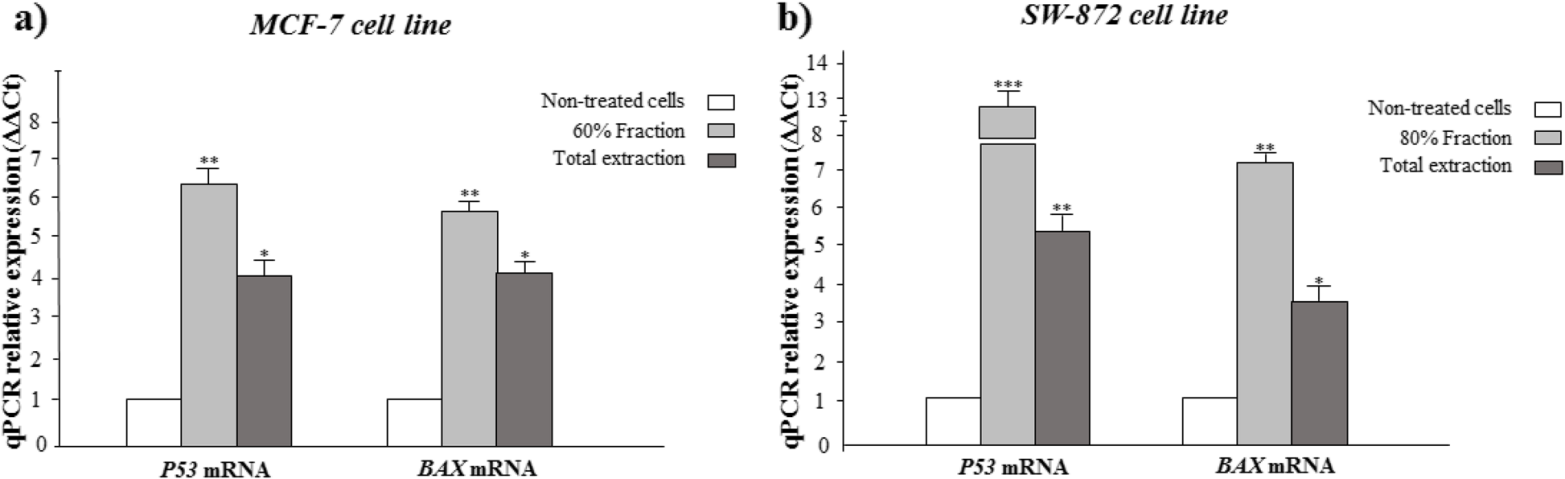
The mRNA expression level of *P53* and *BAX* genes in treated MCF-7 and SW872 cancer cells compared to non-treated cells. Data are mean ± S.E.M. * *P* < 0.05, ** *P* < 0.01

**Fig. 6:**
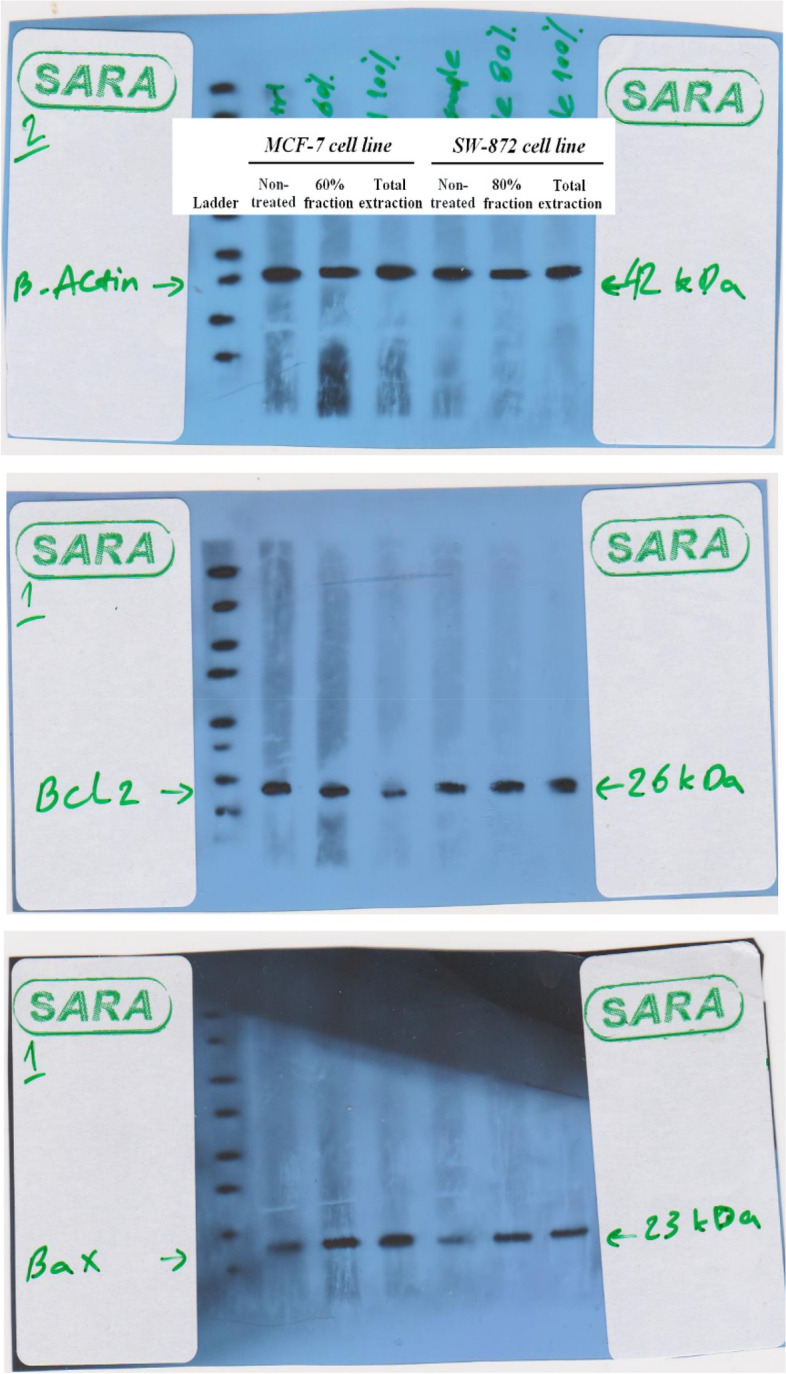
The proteomic levels of BCL-2 and BAX proteins in treated MCF-7 and SW872 cancer cells compared to non-treated cells

To confirm the induction of apoptosis by *A. marschalliana* extract in MCF-7 and SW872 cancer cells, the expression level of mRNA of *P53* and *BAX* genes was investigated. After 24 h of exposure to the determined IC_50_ concentration induced by *A. marschalliana* extract, mRNA levels were evaluated by RT-PCR with related primers. The RT-PCR efficiency of all tests was between 1.82–1.93. The data showed the upregulation of *P53* and *BAX* gene expression as the main pro-apoptotic gene. Although the expression levels of *P53* and *BAX* were significantly increased in treated-cancer cells, they had no significant change in non-treated cells (Fig. 5a and b). Furthermore, an increase in BAX protein level based on the results of western blot analysis is strong evidence to proof of previous test results. In addition, there was a slight decrease in BCL-2 protein expression level in treated-cancer cells; however, the change was not significant (Fig. 6). These findings indicate the potential efficacy of the extract that is mediated by its ability to induce programmed cell death.

#### GC/MS analysis results

GC/MS analysis was performed to obtain the most effective compounds. The results led to obtaining of 45 compounds from the DCM extract. Among the compounds, β-thujone and comphore have the highest amounts including (152.23 g/mol, Retention time = 14.02 min) and (152.23 g/mol, Retention time = 15.05 min), respectively. It seems that these two compounds as the major compounds play an important role in showing the observed effects (Table 3).

**Table 3:** The GC/MS analysis of the identification of *A. marschalliana* DCM (dichloromethane) extract compounds

NO	Compounds	Molecular formula	Molecular mass (g/mol)	Retention time (min)	Percent of area	RIa	RIb
1	Octane	C_8_H_18_	114	3.9	0.67	802	800
2	*Cis*-Salvene	C_9_H_16_	124	5.13	1.03	852	852
3	*α-*Pinene	C_10_H_16_	136	7.47	0.7	933	936
4	Tricyclene	C_10_H_16_	136	7.8	0.74	943	923
5	Camphene	C_10_H_16_	136	7.96	2.50	947	949
6	Sabinene	C_10_H_16_	136	8.83	0.8	973	970
7	*β* -Phellandrene	C_10_H_16_	136	9.93	0.82	1005	1013
8	*α-*Terpinene	C_10_H_16_	136	10.36	1.07	1017	1014
9	*γ*-Terpinen	C_10_H_16_	136.24	11.97	1.81	1061	1062
10	*Trans*-Sabinene hydrate	C_10_H_18_O	154.25	13.17	1.14	1094	1097
11	***β*-Thujone**	**C**_**10**_**H**_**16**_**O**	**152.23**	**14.02**	**8.80**	**1118**	**1116**
12	3-Thujanone	C_10_H_18_O	154.25	14.06	9.40	1119	1112
13	**Camphor**	**C**_**10**_**H**_**16**_**O**	**152.23**	**15.05**	**11.54**	**1146**	**1145**
14	*(E)-p-*2-Menthen-1-ol	C_10_H_18_O	154.25	15.11	1.63	1148	1140
15	*Trans*-Bornyl acetate	C_10_H_16_O	152.23	17.54	2.02	1216	1174
16	2-Methyl-3-phenyl-propanal	C_10_H_12_O	148.2	18.59	1.32	1247	1284
17	*D*-Carvone	C_10_H_14_O	150.22	18.75	1.20	1251	1234
18	Methyl hydrocinnamate	C_10_H_12_O_2_	164.2	19.77	0.74	1281	1286
19	Eugenol	C_10_H_12_O_2_	164.2	22.62	0.76	1367	1355
20	Copaene	C_15_H_24_	204.35	22.97	1.13	1378	1382
21	Methyl cinnamate	C_10_H_10_O_2_	162.18	23.63	0.70	1398	1388
22	Jasmone	C_11_H_16_O	164.24	23.81	0.89	1404	1392
23	*γ-*Gurjunene	C_15_H_24_	204.35	24.05	0.80	1412	1400
24	*p*-Menth-1-en-9-ol	C_10_H_18_O	154.25	26.06	0.64	1476	1294
25	Germacrene D	C_15_H_24_	204.35	26.25	4.70	1483	1480
26	γ -Cadinene	C_15_H_24_	204.35	26.54	0.85	1492	1493
27	Bicyclo germacrene	C_15_H_24_	204.35	26.73	1.46	1498	1494
28	*β*-Gurjenene	C_15_H_24_	204.35	27.02	0.74	1508	1504
29	*α* -Amorphene	C_15_H_24_	204.35	27.08	0.73	1510	1542
30	*σ*-Cadinene	C_15_H_24_	204.35	27.55	0.88	1526	1524
31	(−)-Spathulenol	C_15_H_24_O	220.35	29.22	1.49	1583	1582
32	Caryophyllene oxide	C_15_H_24_O	220.35	29.34	0.94	1587	1583
33	Isoaromadendrene epoxide	C15H24O	220.35	30.17	0.76	1616	1594
34	Ledene	C_15_H_24_	204.35	31.15	0.74	1651	1656
35	Aromadendrene	C_15_H_24_	204.35	31.26	0.69	1655	1449
36	Vulgarol B	C_15_H_24_O	220.35	31.87	0.68	1677	1688
37	Isocaryophyllene	C_15_H_24_	204.35	32.3	0.87	1692	1404
38	Elemol	C_15_H_26_O	222.37	33.75	0.76	1732	1542
39	Naphthalene	C_15_H_24_	204.35	35.15	0.66	1800	1196
40	Valerenol	C_15_H_24_O	220.35	35.48	0.69	1813	1736
41	Hexahydrofarnesyl acetone	C_18_H_36_O	268.5	36.36	0.77	1847	1845
42	Epimanoyl oxide	C_20_H_34_O	290.5	39.95	0.76	1993	2002
43	Eicosane	C_20_H_42_	282.5	40.09	0.68	1999	2000
44	Phytol	C_20_H_40_O	296.5	42.79	1.07	2117	2135
45	*D*-Nerolidol	C15H26O	222	47.05	0.66	2314	2314

RI^a^: Calculation retention index based on the HP-5MS column

RI^b^: Retention index in the literature

## Discussion

Recently, non-communicable diseases such as cancer are considered the most important cause of death in the world. The disease, which is one of the most common and growing diseases, is a major part of the care system’s efforts, with cancer costing the international community [5, 33]. Liposarcoma is the most common soft tissue sarcoma in adults treated with surgery and radiotherapy; however, the metastatic forms of this cancer only respond to chemotherapy, which is accompanied by inadequate side effects and efficacy, resulting in reduced survival rates [34, 35]. Breast cancer is also the most important cause of cancer death among women due to its high prevalence (1 in 4 cases of cancer in women is related to breast cancer) [5]. Severe side effects are the main difficulty of current chemotherapy drugs. Therefore, there is an urgent need for more studies to find suitable alternatives with fewer side effects [36]. Today, a significant portion of these studies target plants and natural plant-derived products. The use of medicinal plants has been considered due to their biological safety. Many plant compounds have been identified as potential anti-cancer and their number is increasing day by day [37, 38]. On the other hand, most anti-cancer drugs used in chemotherapy regimens are of natural origins, such as the alkaloids vinca, taxanes, and epipodophyllotoxins. Therefore, natural compounds are an important source of research and study in the field of the development of anti-cancer drugs [39]. Plants and their active compounds show anti-cancer effects with antioxidant effects, induction of apoptosis, stopping the cell cycle, inhibiting angiogenesis, and eliminating free radicals. This makes researchers and pharmacists use extracts and active compounds of medicinal plants in the production of new anti-cancer drugs [40]. Due to its anti-bacterial, anti-fungal, and disinfecting properties, the species of Artemisia are widely used in the treatment of some diseases such as malaria, hepatitis, cancer, tumors, neuritis, fever, swelling and wound healing [41]. Various reports have been published about the anti-proliferative activity of fractions and extracts of different species of Artemisia [41–43]. The cytotoxic effect is caused by secondary metabolites and other non-metallic compounds involved in the synthesis. Apoptosis as an important defense system controlling cancer is regulated by carcinogenic and cancer-inhibitory genes. In the internal pathway of cell death, mitochondria, and the external pathway, death receptors play a major role. Apoptosis in the internal pathway through caspase 9 and in the external pathway through caspase 8 activates caspase 3 and initiates chromosomal degradation. Studies have shown that the *P53* gene plays an important role in apoptosis. Cancer cells use different molecular mechanisms to suppress the apoptosis pathway. For example, increased expression of BCL-2 (anti-apoptotic protein) or decreased expression or mutation in pro-apoptotic proteins such as BAX [44].

Essential oil of *Artemisia annua* can induce apoptosis of cultured SMMC-7721 cells. Artemisinin, a compound of sweet wormwood (*A. annua*), showed selective toxicity to cancer cells in vitro. In addition, it is given orally to delay the growth of breast cancer in mice treated with 7,12-dimethylbenz [α] anthracene (DMBA) [45, 46]. The ability of smoke and aqueous extracts of *A. princeps* to induce apoptosis was examined on MCF-7 human breast cancer cells in vitro. Smoke and water-soluble extracts induce apoptosis through the mitochondrial pathway in breast cancer cells [16]. In this research, we studied the cytotoxicity of *A. marschalliana* against MCF-7 and SW872 cancer cell lines MCF-10A normal cell line. In this regard, after culturing, cytotoxicity and IC_50_ values were obtained from the treatment of these cells with different extracts of n-Hex, DCM, and MeOH. The plant was evaluated in different concentrations and times. DCM extract showed the strongest cytotoxicity on cancer cells compared to n-Hex and MeOH extracts; therefore, fractionation with the VLC method was carried out for this extract and the cytotoxicity of obtained fractions was evaluated. Because apoptosis or programmed death is one of the natural homeostatic mechanisms of cells in the body and is an important marker in cellular studies for the cytotoxicity of anti-cancer compounds [44], the mechanism of cytotoxicity was evaluated by Flow cytometry annexin V/PI kit. According to our finding, the occurrence of cytotoxic effects depends on the concentration of extracts and fractions (*p* < 0.05) and the increase of the plant samples concentration causes to decrease in the cell viability. In the MCF-7 cell line, the cell viability with treated DCM extract and the mentioned fractions depends on the time of exposure (*p* < 0.001). Regarding time-dependent effects, it can be argued that the active ingredients may be changed from prodrug to active drug over time of incubation and show cytotoxic effects, or after participating in intracellular metabolic pathways, the active metabolite is more than the main drug. Based on the apoptosis analysis, the rate of apoptosis is the highest value for DCM extract, 60 and 80% fractions in SW872 cell line and 60% fraction in MCF-7 cell. Statistical calculation results illustrated that the viability of MCF-7 cell line, in the face of 60% fraction was significantly different (*p* < 0.0001) from other fractions. It can be concluded that the fraction inhibits 60% of cancer cell growth by inducing apoptosis. The cytotoxic content of this fraction showed the highest rate of apoptosis among the other (43.6%). Moreover, the viability of the 60% fraction decreases over time and the effects of cytotoxicity are time dependent (*P* < 0.001). The viability of the 60% fraction in the incubation times of 24 and 48 hours, in this cell line was significantly different from the MCF-10A cell line (*p* < 0.05), which indicates the selective cytotoxic effects in the MCF-7 cell line. Comparison of IC_50_ values indicated that 40, 80, and 100% fractions after 48 hours of incubation significantly inhibited the growth of cancer cells, but the mechanism of cytotoxicity of these fractions is from the necrosis pathway. Therefore, in this cell line, 60% fraction is a suitable candidate for additional studies. Statistical analyzes in the SW872 cell line indicated that the viability of the most cytotoxic fraction with the lowest IC_50_ values (60 and 80% fractions) was significantly different (*p* < 0.0001) with 40 and 100% fractions. Our finding showed that the 60 and 80% fractions inhibited cell growth by apoptosis induction pathway. In addition, the percentage of apoptotic cells in the 80% fraction is higher than the 60% fraction. The viability of 60 and 80% fractions are significantly different from DCM extract (*p* < 0.05) without depending on the time. A comparison of 80% fraction viability at both incubation times (24 and 48 h) showed that the cytotoxic effects of 80% fraction in SW872 cells compared to normal cells with a *p*-value less than 0.05 were selective. Thus, in total, 80% fraction is a better candidate for future research on liposarcoma cancer cells in future research. However, it is possible to obtain compounds that have selective cytotoxic effects on cancer cells by purifying the cytotoxic active ingredients from the 60% fraction. The results of the MTT test and statistical analysis showed that 60 and 80% fractions probably contain the most effective cytotoxic compounds against MCF-7 and SW872 cancer cells. The cytotoxic effects of these fractions are selectively on cancer cell lines with fewer adverse effects on the normal cell line. The essential oil also significantly inhibited cell growth. It has been demonstrated that the DCM extract of *A. turanica* had significant anti-cancer effects against K562 and HL-60 cell lines [47]. Two triterpenoid compounds called lopeol and lodartin were purified from *A. indica* as a cytotoxic active ingredient. These compounds inhibited the growth of breast cancer cells with IC_50_ values ​​of 28.08 and 25.18 μM [48]. The antioxidant and anti-cancer properties of *A. marschalliana* ethanolic extract were investigated and AGS trans phytol (29.22%), alpha-linoleic acid (13.47%), and n-hexadecanoic acid (9/28%) were reported as the effective cytotoxic compounds [49]. On the other hand, the anti-cancer effects of silver nanoparticles synthesized by *A. marschalliana* shoot against human gastrointestinal carcinoma were studied and flow cytometry results showed that the cytotoxic effects of silver nanoparticles with IC_50_ are equivalent to 21.05 μg/mL due to induction of apoptosis [50]. Based on previous studies, the DCM extract of *Artemisia* has significant cytotoxic effects due to the presence of effective anti-cancer compounds. β-thujone and comphore were detected as the major compounds that the effective compounds according to the GC-MS analysis. Some of the antitumor agents extracted from Artemisia species include cesquiterpenalactones, terpenoids and flavonoids [34]. As previously mentioned, flow cytometry results illustrated that this extract leads the cell to apoptosis. Analysis of pro-apoptotic gene expression levels by RT-PCR showed that *A. marschalliana* extracts increased *P53* and *BAX* expression. In addition, the increase in BAX proteins level supported the RT-PCR data. These findings confirm the involvement of the innate pathway of cell death and suggest that *A. marschalliana* extracts induce apoptosis in cancerous cells. According to the findings, it can be concluded that 60 and 80% fractions of DCM extract of *A. marschalliana* can be a potential option for future studies in the field of identification and separation of pure cytotoxic compounds.

## Conclusion

Our results showed that the cytotoxic effects of *A. marschalliana* shoots are related to DCM extract. The 60% fraction has the highest effects of cytotoxicity by the mechanism of apoptosis in the MCF-7 cell line. It seems that this fraction has selective toxicity on cancer cells and can prohibit their cell growth, which increases with the incubation time. The viability of SW872 cells treated with 60 and 80% fractions of DCM extract was significantly reduced with other fractions. The main mechanism of cytotoxicity of 60 and 80% fractions in SW872 cells was the induction of apoptosis and 80% fraction selectively inhibited the growth of cancer cells. The essential oil of this plant also inhibited the growth of MCF-7 cancer cells. According to the above findings, it can be argued that the cell growth inhibitory compounds of DCM extract have accumulated in 60 and 80% fractions. Therefore, 60 and 80% fractions of DCM extract are the best candidates for future studies in the field of identification and separation of pure cytotoxic compounds.

## Data Availability

The datasets used and/or analyzed during the current study are available from the corresponding author upon reasonable request.

## Abbreviations

RT-PCR: Real-Time Polymerase chain reaction
BMI: Body mass index
LS: Liposarcoma
n-Hex: n-Hexane
DCM: Dichloromethane
MeOH: methanol
MCF-7 cell: Human breast adenocarcinoma cell line
SW872 cell: Human liposarcoma cell line
MCF-10A cell: Normal human mammary epithelial cells
REC: Research Ethical Committee
DMSO: Dimethyl sulfoxide
DOS: Desoxycholic acid sodium salt
IC50: Half-maximal inhibitory concentration
